# Is It the Chinese Plague?

**Published:** 1919-01

**Authors:** 


### Is It the Chinese Plague?

Is influenza identified with the "Chinese pneumonia plague" of 1910? There has been much difficulty in locating the origin of the present epidemic. Dr. James Joseph King of the United States Army Medical Corps, says that the symptoms of the two diseases are similar and that some of them are different from those observed in previous influenza epidemics. He thinks that Chinese coolies working in the trenches in France took the disease germs there and asserts that wherever numbers of Chinese laborers have gone since 1910 they have carried these pneumonia germs which are in fact groups of different germs. He regards the name by which the disease is known as a misnomer. The disease first appeared in the German lines, the plague bacilli being distributed, Dr. King thinks, by captured Chinese laborers. Dr. King concludes: "There is a striking similarity between the pneumonic plague of North China and the present so-called Spanish influenza epidemic. It is not unreasonable to believe that the two diseases may be the same. The influenza baccillus and the bacillus pestis (plague bacillus), in atypical forms, may simulate each other. We know that organisms may assume different conditions. The ordinary influenza bacillus is a short, slender bacillus. The plague bacillus is about the same length, but is generally fatter and broader. * * * It seems possible that the bacillus pestis may have been present in a non-virulent state in the Chinese coolies, and assumed new virulence, vigor and a somewhat different form, when transplanted into virgin soil. The high mortality and infectivity of this epidemic strongly suggest it.

"On this basis the epidemics which have followed all great wars may be explained. If a nation or tribe can survive any disease long enough it will acquire immunity to that disease. When, however, foreign people commingle freely and intimately, as in war, epidemics will become virulent in some other race which has not acquired immunity to that specific organism."

---

Would you let a child starve to death? You can help save 400,000 children by subscribing to the Armenian Relief Campaign, February 3-10.

